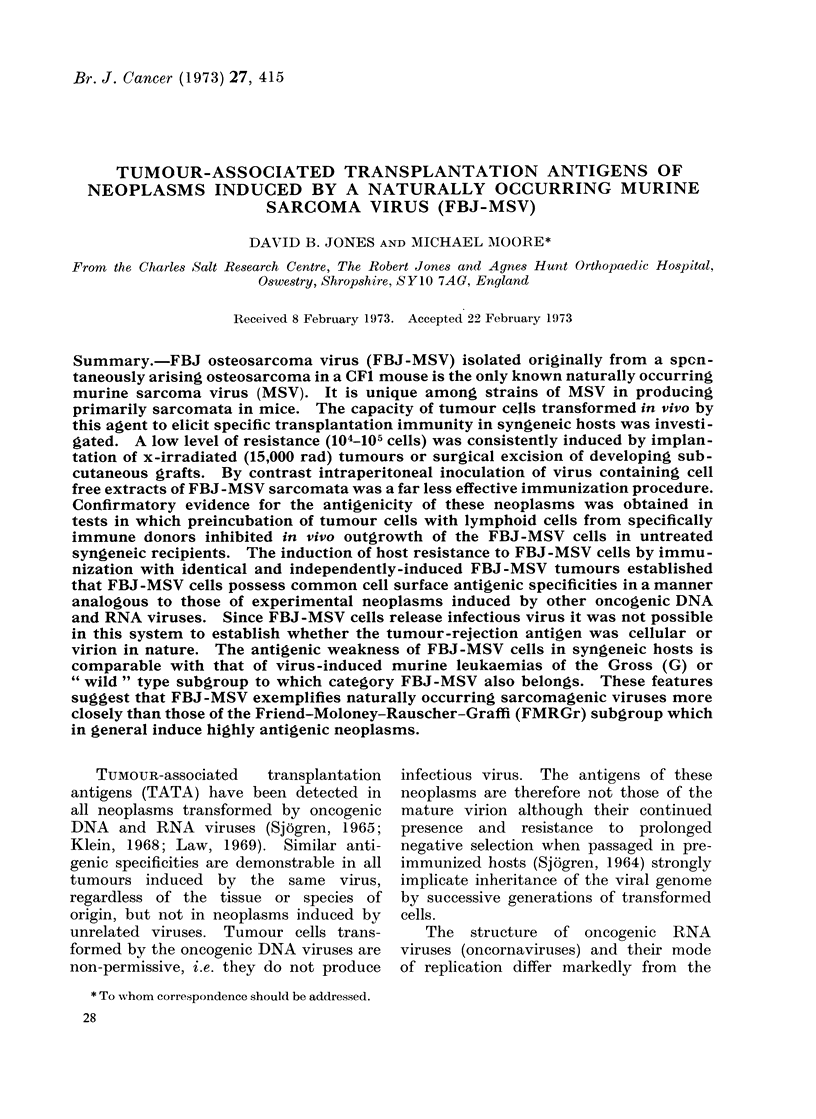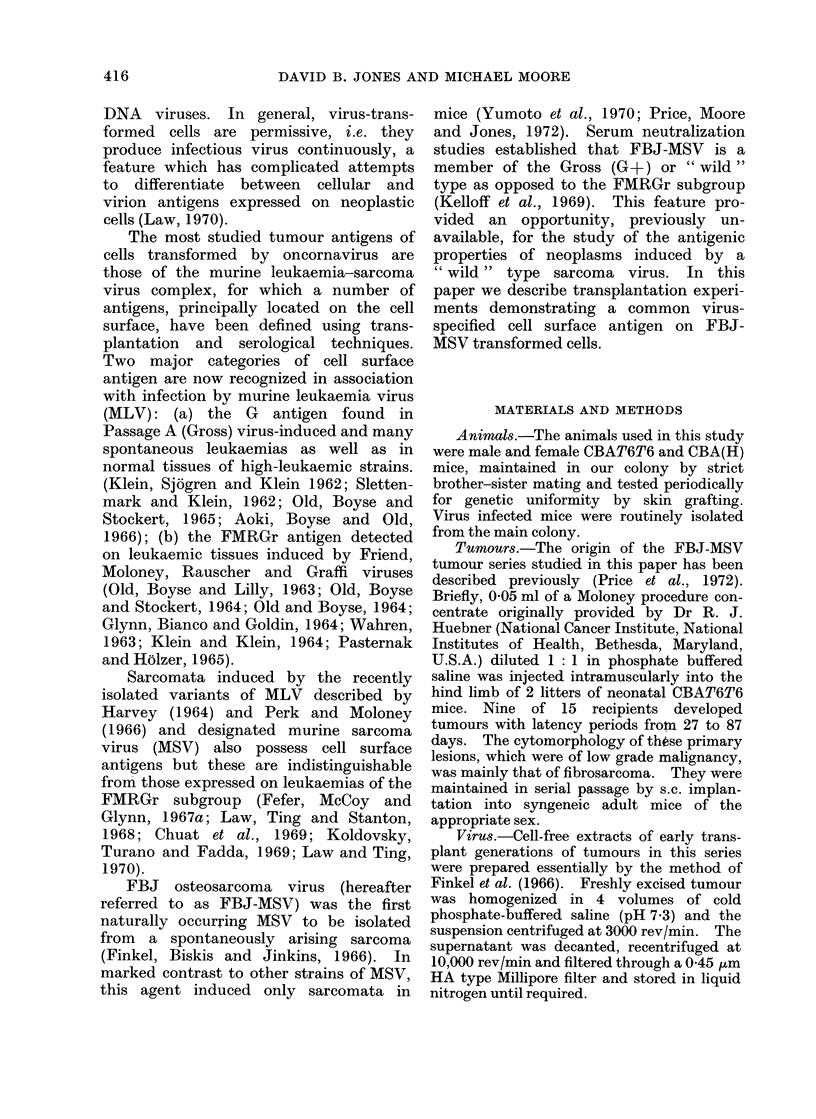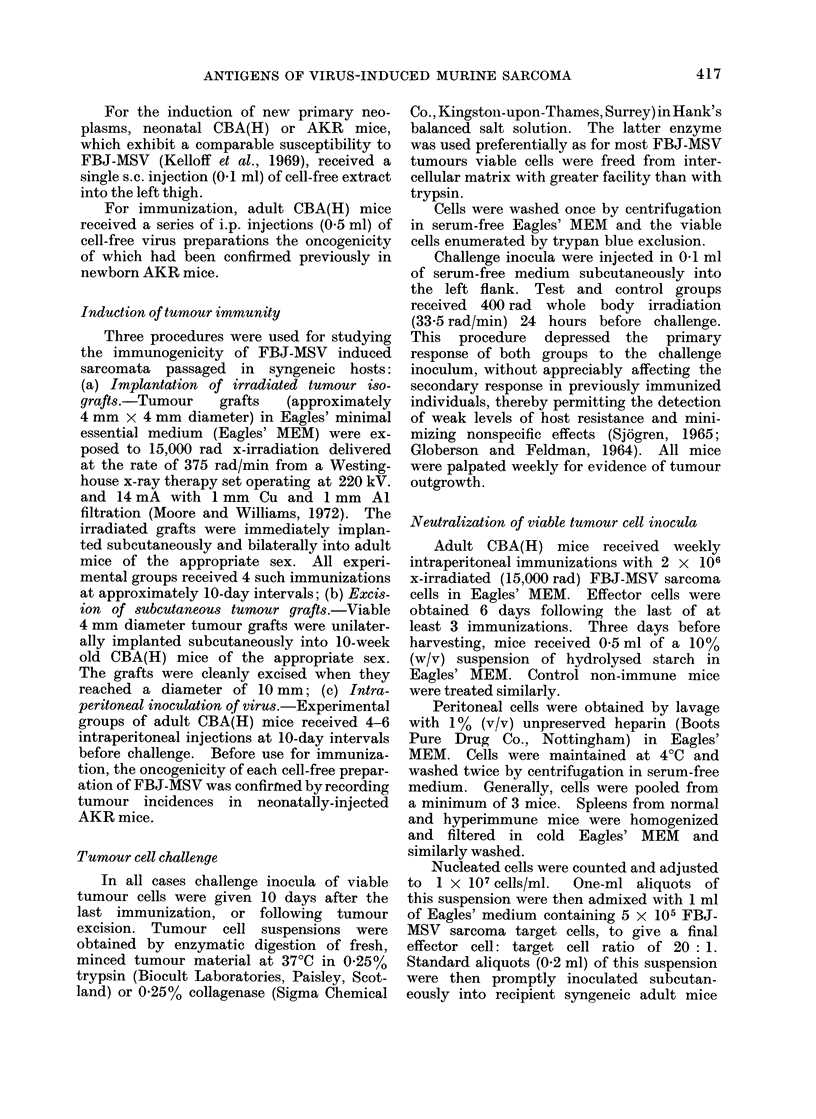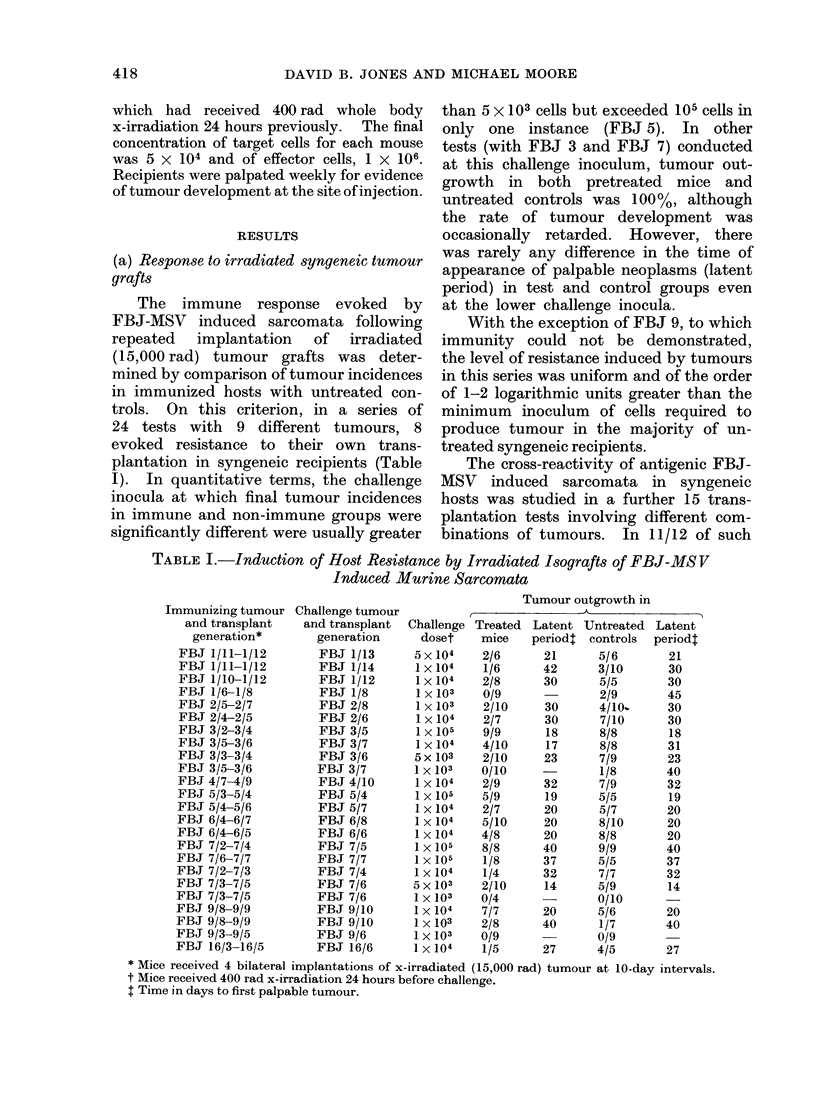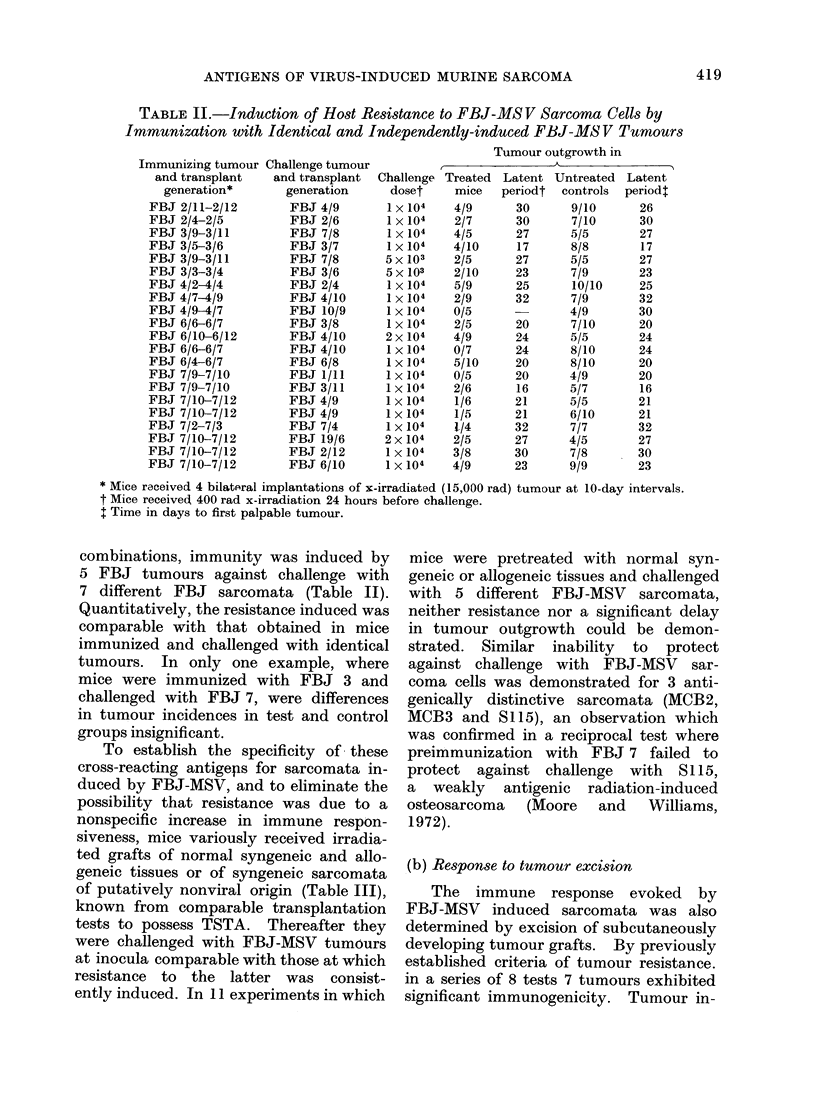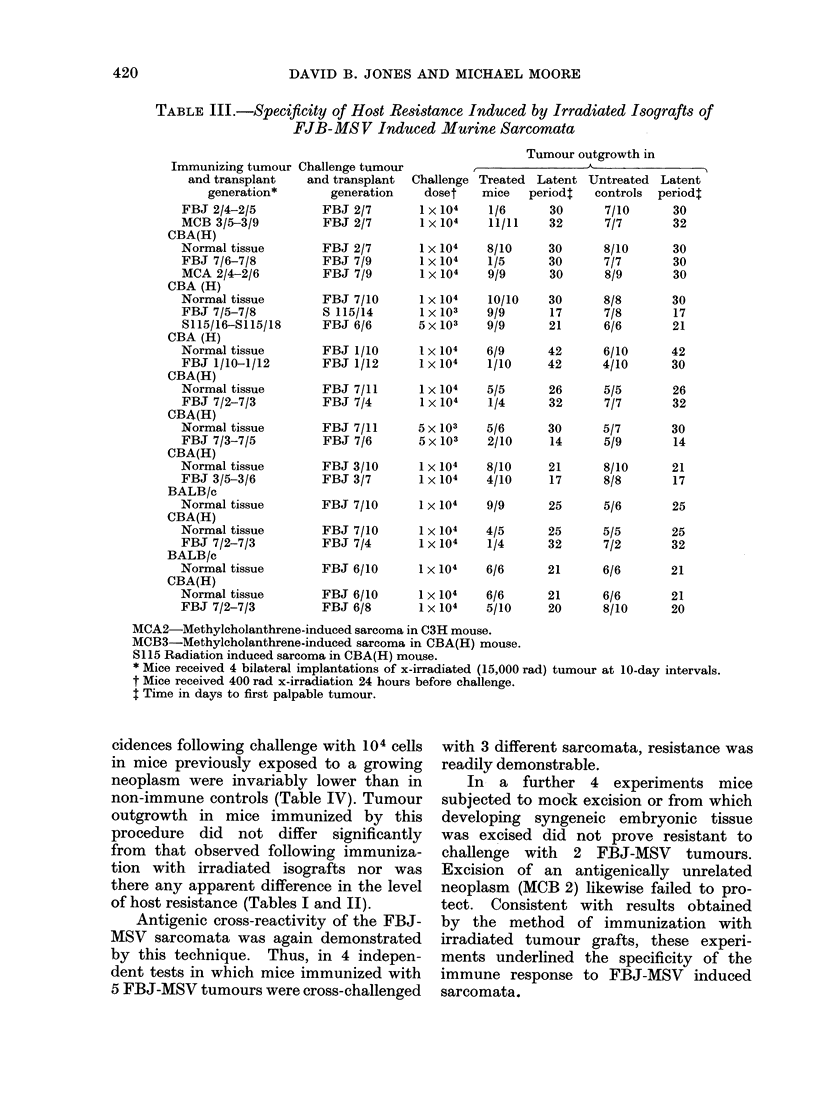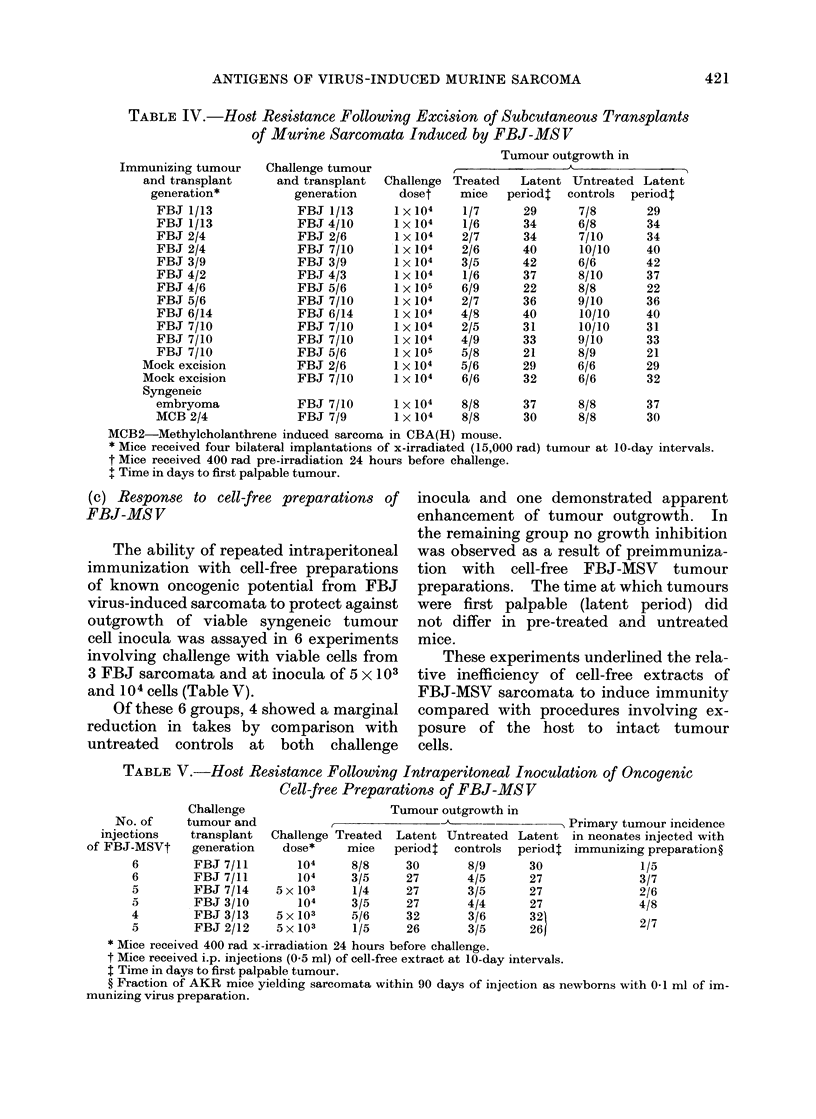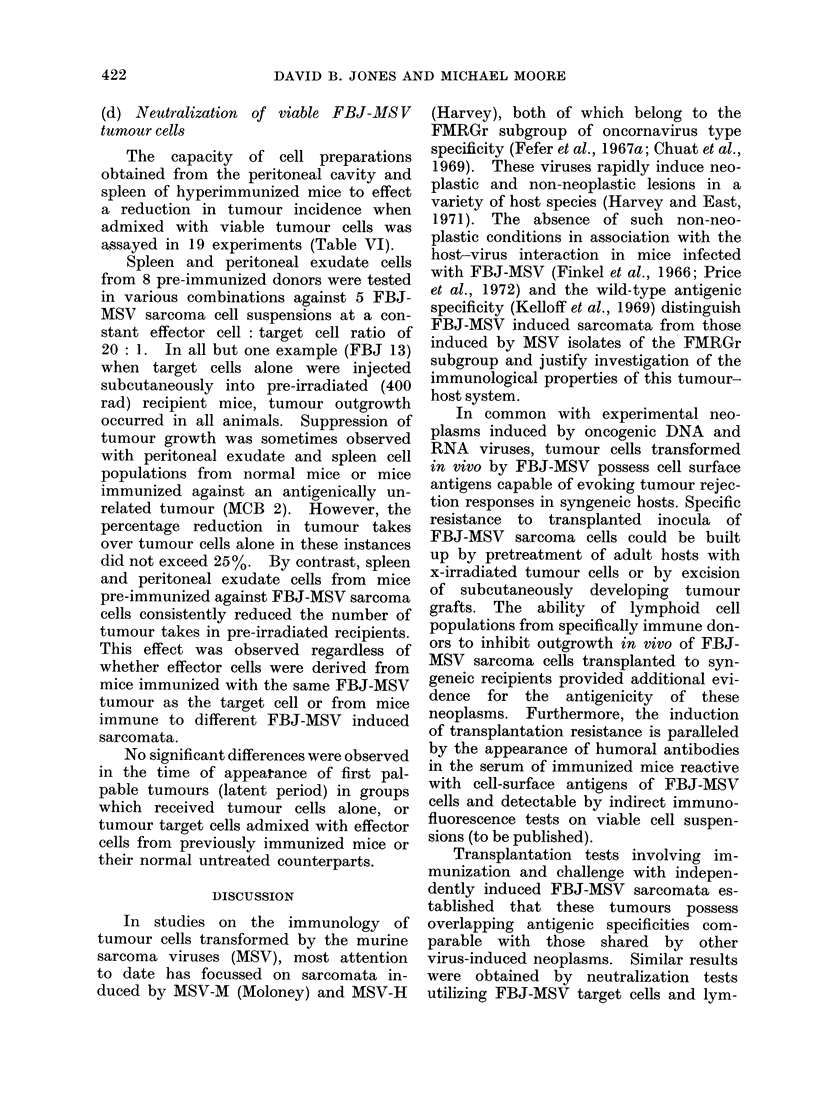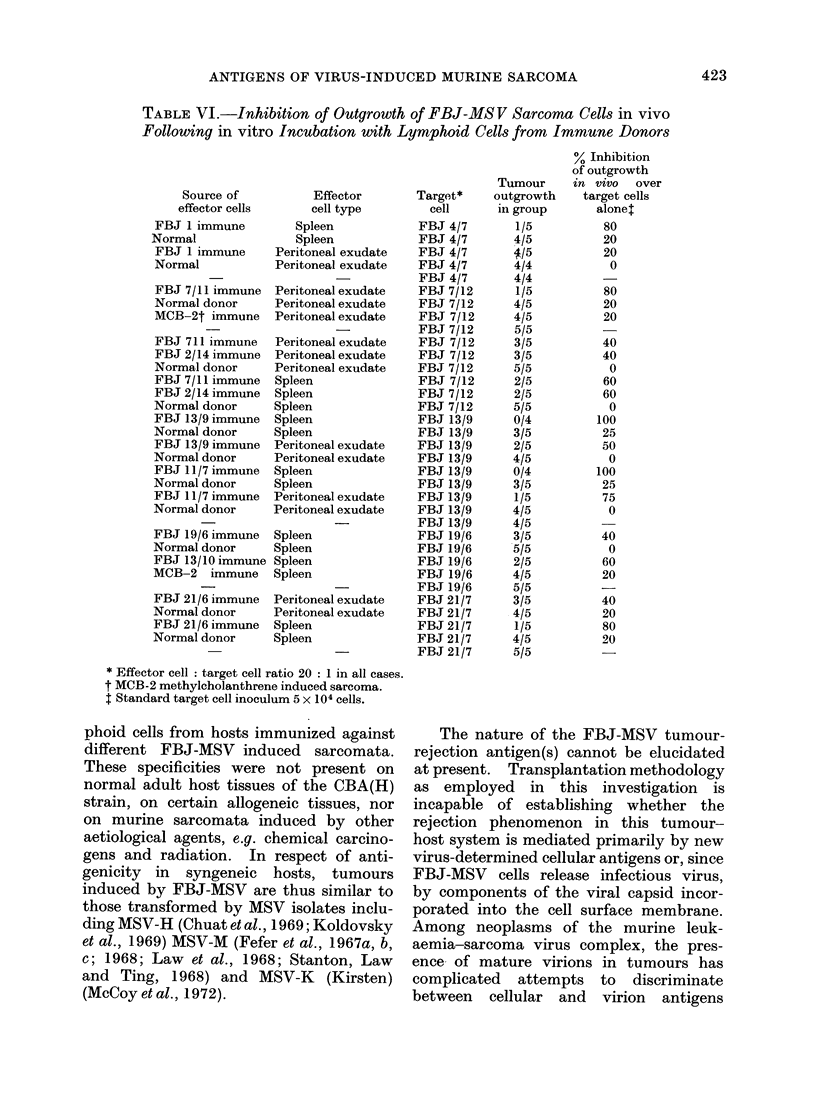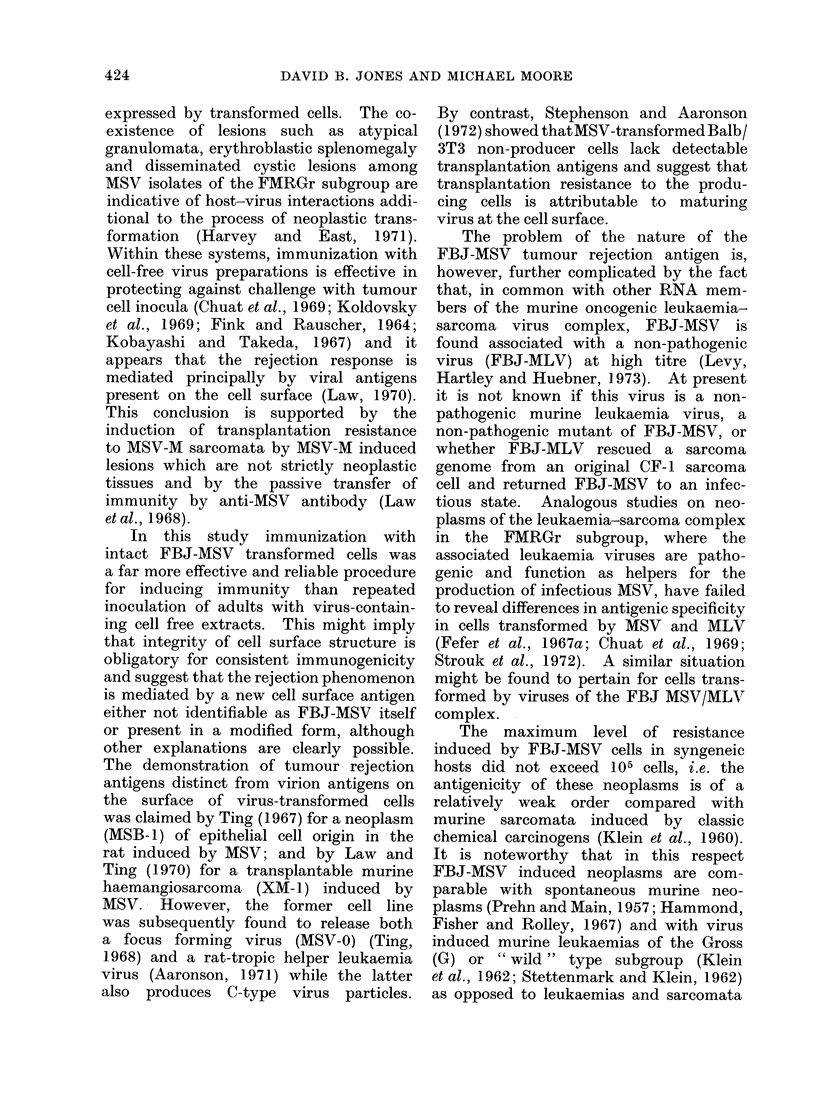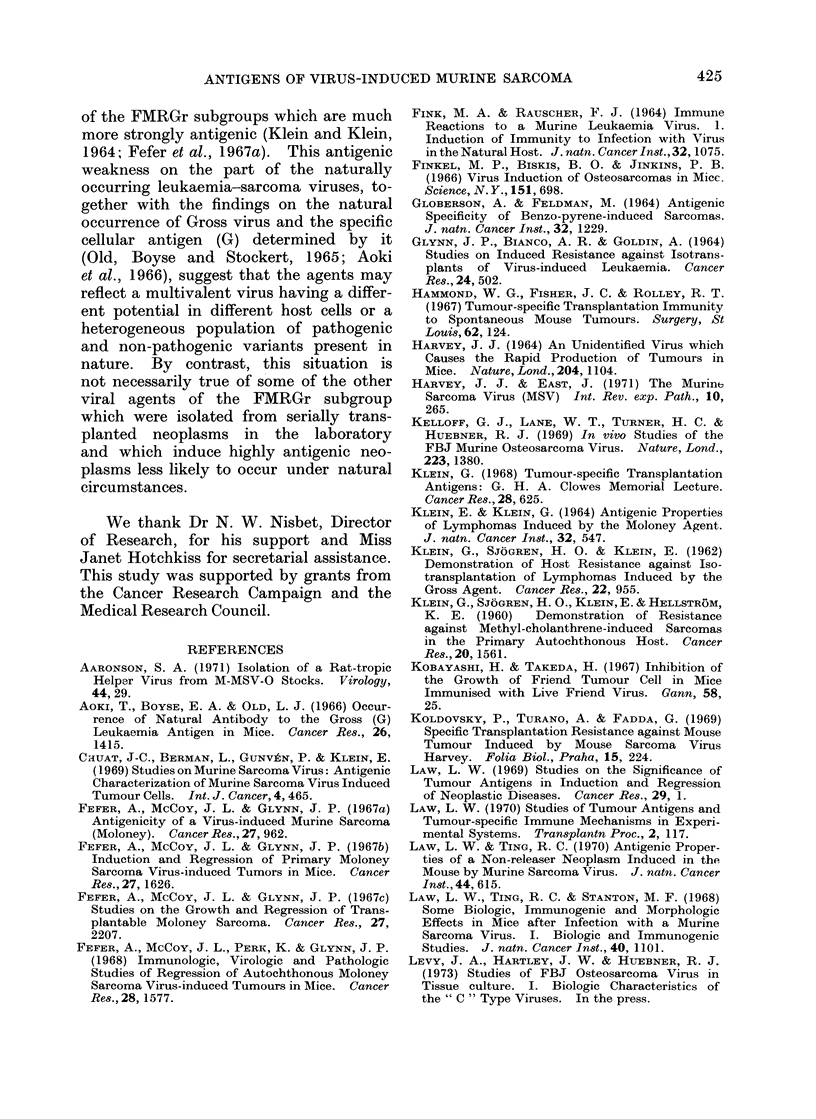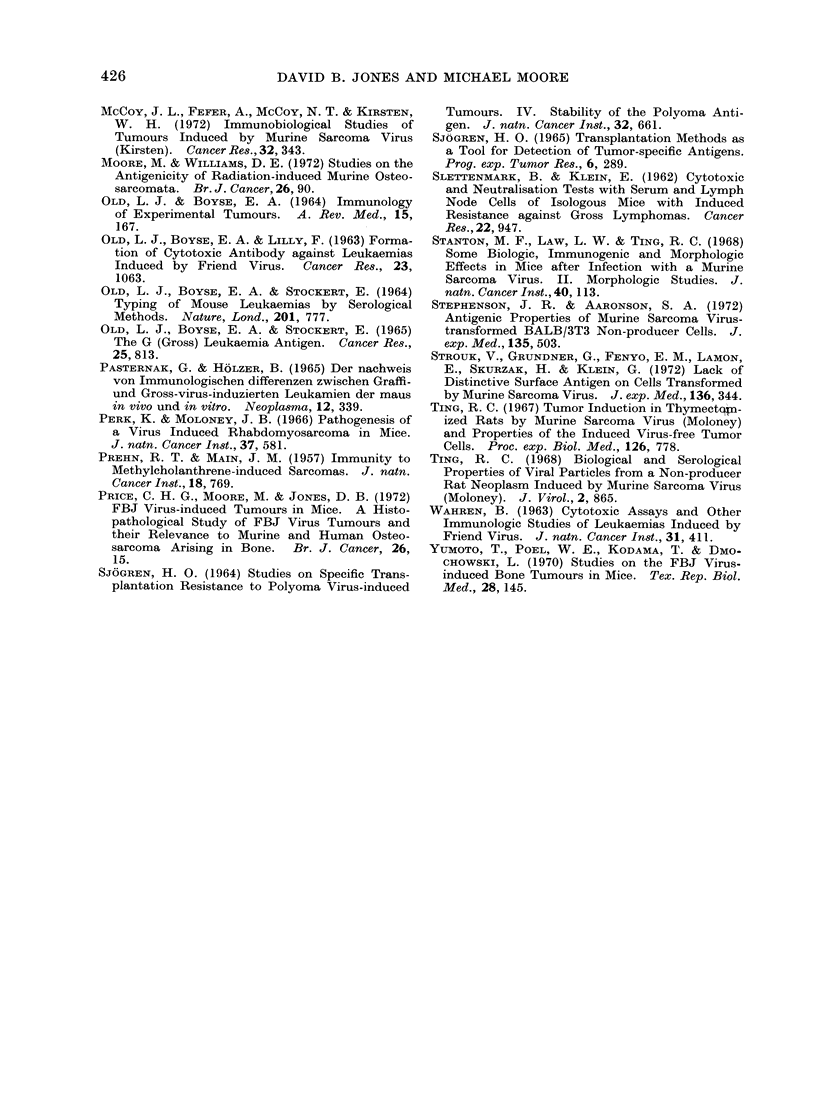# Tumour-Associated Transplantation Antigens of Neoplasms Induced by a Naturally Occurring Murine Sarcoma Virus (FBJ-MSV)

**DOI:** 10.1038/bjc.1973.52

**Published:** 1973-06

**Authors:** David B. Jones, Michael Moore

## Abstract

FBJ osteosarcoma virus (FBJ-MSV) isolated originally from a spontaneously arising osteosarcoma in a CF1 mouse is the only known naturally occurring murine sarcoma virus (MSV). It is unique among strains of MSV in producing primarily sarcomata in mice. The capacity of tumour cells transformed *in vivo* by this agent to elicit specific transplantation immunity in syngeneic hosts was investigated. A low level of resistance (10^4^-10^5^ cells) was consistently induced by implantation of x-irradiated (15,000 rad) tumours or surgical excision of developing subcutaneous grafts. By contrast intraperitoneal inoculation of virus containing cellfree extracts of FBJ-MSV sarcomata was a far less effective immunization procedure. Confirmatory evidence for the antigenicity of these neoplasms was obtained in tests in which preincubation of tumour cells with lymphoid cells from specifically immune donors inhibited *in vivo* outgrowth of the FBJ-MSV cells in untreated syngeneic recipients. The induction of host resistance to FBJ-MSV cells by immunization with identical and independently-induced FBJ-MSV tumours established that FBJ-MSV cells possess common cell surface antigenic specificities in a manner analogous to those of experimental neoplasms induced by other oncogenic DNA and RNA viruses. Since FBJ-MSV cells release infectious virus it was not possible in this system to establish whether the tumour-rejection antigen was cellular or virion in nature. The antigenic weakness of FBJ-MSV cells in syngeneic hosts is comparable with that of virus-induced murine leukaemias of the Gross (G) or “wild” type subgroup to which category FBJ-MSV also belongs. These features suggest that FBJ-MSV exemplifies naturally occurring sarcomagenic viruses more closely than those of the Friend-Moloney-Rauscher-Graffi (FMRGr) subgroup which in general induce highly antigenic neoplasms.


Br. J. Cancer (1973) 27, 415

### TUMOUR-ASSOCIATED TRANSPLANTATION ANTIGENS OF NEOPLASMS INDUCED BY A NATURALLY OCCURRING MURINE SARCOMA VIRUS (FBJ-MSV)

DAVID B. JONES AND MICHAEL MOORE*

*From the Charles Salt Research Centre, The Robert Jones and Agnes Hunt Orthopaedic Hospital, Oswestry, Shropshire, SY10 7AG, England*

Received 8 February 1973. Accepted 22 February 1973

**Summary.—FBJ osteosarcoma virus (FBJ-MSV) isolated originally from a spontaneously arising osteosarcoma in a CF1 mouse is the only known naturally occurring murine sarcoma virus (MSV). It is unique among strains of MSV in producing primarily sarcomata in mice. The capacity of tumour cells transformed *in vivo* by this agent to elicit specific transplantation immunity in syngeneic hosts was investigated. A low level of resistance ($10^4$–$10^5$ cells) was consistently induced by implantation of x-irradiated (15,000 rad) tumours or surgical excision of developing subcutaneous grafts. By contrast intraperitoneal inoculation of virus containing cell free extracts of FBJ-MSV sarcomata was a far less effective immunization procedure. Confirmatory evidence for the antigenicity of these neoplasms was obtained in tests in which preincubation of tumour cells with lymphoid cells from specifically immune donors inhibited *in vivo* outgrowth of the FBJ-MSV cells in untreated syngeneic recipients. The induction of host resistance to FBJ-MSV cells by immunization with identical and independently-induced FBJ-MSV tumours established that FBJ-MSV cells possess common cell surface antigenic specificities in a manner analogous to those of experimental neoplasms induced by other oncogenic DNA and RNA viruses. Since FBJ-MSV cells release infectious virus it was not possible in this system to establish whether the tumour-rejection antigen was cellular or virion in nature. The antigenic weakness of FBJ-MSV cells in syngeneic hosts is comparable with that of virus-induced murine leukaemias of the Gross (G) or " wild " type subgroup to which category FBJ-MSV also belongs. These features suggest that FBJ-MSV exemplifies naturally occurring sarcomagenic viruses more closely than those of the Friend–Moloney–Rauscher–Graffi (FMRGr) subgroup which in general induce highly antigenic neoplasms.**

TUMOUR-associated transplantation antigens (TATA) have been detected in all neoplasms transformed by oncogenic DNA and RNA viruses (Sjögren, 1965; Klein, 1968; Law, 1969). Similar antigenic specificities are demonstrable in all tumours induced by the same virus, regardless of the tissue or species of origin, but not in neoplasms induced by unrelated viruses. Tumour cells transformed by the oncogenic DNA viruses are non-permissive, *i.e.* they do not produce infectious virus. The antigens of these neoplasms are therefore not those of the mature virion although their continued presence and resistance to prolonged negative selection when passaged in pre-immunized hosts (Sjögren, 1964) strongly implicate inheritance of the viral genome by successive generations of transformed cells.

The structure of oncogenic RNA viruses (oncornaviruses) and their mode of replication differ markedly from the

* To whom correspondence should be addressed.

DNA viruses. In general, virus-transformed cells are permissive, *i.e.* they produce infectious virus continuously, a feature which has complicated attempts to differentiate between cellular and virion antigens expressed on neoplastic cells (Law, 1970).

The most studied tumour antigens of cells transformed by oncornavirus are those of the murine leukaemia–sarcoma virus complex, for which a number of antigens, principally located on the cell surface, have been defined using transplantation and serological techniques. Two major categories of cell surface antigen are now recognized in association with infection by murine leukaemia virus (MLV): (a) the G antigen found in Passage A (Gross) virus-induced and many spontaneous leukaemias as well as in normal tissues of high-leukaemic strains. (Klein, Sjögren and Klein 1962; Slettenmark and Klein, 1962; Old, Boyse and Stockert, 1965; Aoki, Boyse and Old, 1966); (b) the FMRGr antigen detected on leukaemic tissues induced by Friend, Moloney, Rauscher and Graffi viruses (Old, Boyse and Lilly, 1963; Old, Boyse and Stockert, 1964; Old and Boyse, 1964; Glynn, Bianco and Goldin, 1964; Wahren, 1963; Klein and Klein, 1964; Pasternak and Hölzer, 1965).

Sarcomata induced by the recently isolated variants of MLV described by Harvey (1964) and Perk and Moloney (1966) and designated murine sarcoma virus (MSV) also possess cell surface antigens but these are indistinguishable from those expressed on leukaemias of the FMRGr subgroup (Fefer, McCoy and Glynn, 1967*a*; Law, Ting and Stanton, 1968; Chuat *et al.*, 1969; Koldovsky, Turano and Fadda, 1969; Law and Ting, 1970).

FBJ osteosarcoma virus (hereafter referred to as FBJ-MSV) was the first naturally occurring MSV to be isolated from a spontaneously arising sarcoma (Finkel, Biskis and Jinkins, 1966). In marked contrast to other strains of MSV, this agent induced only sarcomata in mice (Yumoto *et al.*, 1970; Price, Moore and Jones, 1972). Serum neutralization studies established that FBJ-MSV is a member of the Gross (G+) or "wild" type as opposed to the FMRGr subgroup (Kelloff *et al.*, 1969). This feature provided an opportunity, previously unavailable, for the study of the antigenic properties of neoplasms induced by a "wild" type sarcoma virus. In this paper we describe transplantation experiments demonstrating a common virus-specified cell surface antigen on FBJ-MSV transformed cells.

### MATERIALS AND METHODS

*Animals*.—The animals used in this study were male and female CBA*T*6*T*6 and CBA(H) mice, maintained in our colony by strict brother–sister mating and tested periodically for genetic uniformity by skin grafting. Virus infected mice were routinely isolated from the main colony.

*Tumours*.—The origin of the FBJ-MSV tumour series studied in this paper has been described previously (Price *et al.*, 1972). Briefly, 0·05 ml of a Moloney procedure concentrate originally provided by Dr R. J. Huebner (National Cancer Institute, National Institutes of Health, Bethesda, Maryland, U.S.A.) diluted 1 : 1 in phosphate buffered saline was injected intramuscularly into the hind limb of 2 litters of neonatal CBA*T*6*T*6 mice. Nine of 15 recipients developed tumours with latency periods from 27 to 87 days. The cytomorphology of these primary lesions, which were of low grade malignancy, was mainly that of fibrosarcoma. They were maintained in serial passage by s.c. implantation into syngeneic adult mice of the appropriate sex.

*Virus*.—Cell-free extracts of early transplant generations of tumours in this series were prepared essentially by the method of Finkel *et al.* (1966). Freshly excised tumour was homogenized in 4 volumes of cold phosphate-buffered saline (pH 7·3) and the suspension centrifuged at 3000 rev/min. The supernatant was decanted, recentrifuged at 10,000 rev/min and filtered through a 0·45 $\mu$m HA type Millipore filter and stored in liquid nitrogen until required.

For the induction of new primary neoplasms, neonatal CBA(H) or AKR mice, which exhibit a comparable susceptibility to FBJ-MSV (Kelloff *et al.*, 1969), received a single s.c. injection (0·1 ml) of cell-free extract into the left thigh.

For immunization, adult CBA(H) mice received a series of i.p. injections (0·5 ml) of cell-free virus preparations the oncogenicity of which had been confirmed previously in newborn AKR mice.

*Induction of tumour immunity*

Three procedures were used for studying the immunogenicity of FBJ-MSV induced sarcomata passaged in syngeneic hosts: (a) *Implantation of irradiated tumour isografts.*—Tumour grafts (approximately 4 mm × 4 mm diameter) in Eagles' minimal essential medium (Eagles' MEM) were exposed to 15,000 rad x-irradiation delivered at the rate of 375 rad/min from a Westinghouse x-ray therapy set operating at 220 kV. and 14 mA with 1 mm Cu and 1 mm Al filtration (Moore and Williams, 1972). The irradiated grafts were immediately implanted subcutaneously and bilaterally into adult mice of the appropriate sex. All experimental groups received 4 such immunizations at approximately 10-day intervals; (b) *Excision of subcutaneous tumour grafts.*—Viable 4 mm diameter tumour grafts were unilaterally implanted subcutaneously into 10-week old CBA(H) mice of the appropriate sex. The grafts were cleanly excised when they reached a diameter of 10 mm; (c) *Intraperitoneal inoculation of virus.*—Experimental groups of adult CBA(H) mice received 4–6 intraperitoneal injections at 10-day intervals before challenge. Before use for immunization, the oncogenicity of each cell-free preparation of FBJ-MSV was confirmed by recording tumour incidences in neonatally-injected AKR mice.

*Tumour cell challenge*

In all cases challenge inocula of viable tumour cells were given 10 days after the last immunization, or following tumour excision. Tumour cell suspensions were obtained by enzymatic digestion of fresh, minced tumour material at 37°C in 0·25% trypsin (Biocult Laboratories, Paisley, Scotland) or 0·25% collagenase (Sigma Chemical Co., Kingston-upon-Thames, Surrey) in Hank's balanced salt solution. The latter enzyme was used preferentially as for most FBJ-MSV tumours viable cells were freed from intercellular matrix with greater facility than with trypsin.

Cells were washed once by centrifugation in serum-free Eagles' MEM and the viable cells enumerated by trypan blue exclusion.

Challenge inocula were injected in 0·1 ml of serum-free medium subcutaneously into the left flank. Test and control groups received 400 rad whole body irradiation (33·5 rad/min) 24 hours before challenge. This procedure depressed the primary response of both groups to the challenge inoculum, without appreciably affecting the secondary response in previously immunized individuals, thereby permitting the detection of weak levels of host resistance and minimizing nonspecific effects (Sjögren, 1965; Globerson and Feldman, 1964). All mice were palpated weekly for evidence of tumour outgrowth.

*Neutralization of viable tumour cell inocula*

Adult CBA(H) mice received weekly intraperitoneal immunizations with $2 \times 10^6$ x-irradiated (15,000 rad) FBJ-MSV sarcoma cells in Eagles' MEM. Effector cells were obtained 6 days following the last of at least 3 immunizations. Three days before harvesting, mice received 0·5 ml of a 10% (w/v) suspension of hydrolysed starch in Eagles' MEM. Control non-immune mice were treated similarly.

Peritoneal cells were obtained by lavage with 1% (v/v) unpreserved heparin (Boots Pure Drug Co., Nottingham) in Eagles' MEM. Cells were maintained at 4°C and washed twice by centrifugation in serum-free medium. Generally, cells were pooled from a minimum of 3 mice. Spleens from normal and hyperimmune mice were homogenized and filtered in cold Eagles' MEM and similarly washed.

Nucleated cells were counted and adjusted to $1 \times 10^7$ cells/ml. One-ml aliquots of this suspension were then admixed with 1 ml of Eagles' medium containing $5 \times 10^5$ FBJ-MSV sarcoma target cells, to give a final effector cell: target cell ratio of 20 : 1. Standard aliquots (0·2 ml) of this suspension were then promptly inoculated subcutaneously into recipient syngeneic adult mice which had received 400 rad whole body x-irradiation 24 hours previously. The final concentration of target cells for each mouse was $5 \times 10^4$ and of effector cells, $1 \times 10^6$. Recipients were palpated weekly for evidence of tumour development at the site of injection.

### RESULTS

### (a) *Response to irradiated syngeneic tumour grafts*

The immune response evoked by FBJ-MSV induced sarcomata following repeated implantation of irradiated (15,000 rad) tumour grafts was determined by comparison of tumour incidences in immunized hosts with untreated controls. On this criterion, in a series of 24 tests with 9 different tumours, 8 evoked resistance to their own transplantation in syngeneic recipients (Table I). In quantitative terms, the challenge inocula at which final tumour incidences in immune and non-immune groups were significantly different were usually greater than $5 \times 10^3$ cells but exceeded $10^5$ cells in only one instance (FBJ 5). In other tests (with FBJ 3 and FBJ 7) conducted at this challenge inoculum, tumour outgrowth in both pretreated mice and untreated controls was 100%, although the rate of tumour development was occasionally retarded. However, there was rarely any difference in the time of appearance of palpable neoplasms (latent period) in test and control groups even at the lower challenge inocula.

With the exception of FBJ 9, to which immunity could not be demonstrated, the level of resistance induced by tumours in this series was uniform and of the order of 1–2 logarithmic units greater than the minimum inoculum of cells required to produce tumour in the majority of untreated syngeneic recipients.

The cross-reactivity of antigenic FBJ-MSV induced sarcomata in syngeneic hosts was studied in a further 15 transplantation tests involving different combinations of tumours. In 11/12 of such

TABLE I.—*Induction of Host Resistance by Irradiated Isografts of FBJ-MSV Induced Murine Sarcomata*

| Immunizing tumour and transplant generation* | Challenge tumour and transplant generation | Challenge dose† | Tumour outgrowth in: Treated mice | Latent period‡ | Untreated controls | Latent period‡ |
|---|---|---|---|---|---|---|
| FBJ 1/11–1/12 | FBJ 1/13 | $5 \times 10^4$ | 2/6 | 21 | 5/6 | 21 |
| FBJ 1/11–1/12 | FBJ 1/14 | $1 \times 10^4$ | 1/6 | 42 | 3/10 | 30 |
| FBJ 1/10–1/12 | FBJ 1/12 | $1 \times 10^4$ | 2/8 | 30 | 5/5 | 30 |
| FBJ 1/6–1/8 | FBJ 1/8 | $1 \times 10^3$ | 0/9 | — | 2/9 | 45 |
| FBJ 2/5–2/7 | FBJ 2/8 | $1 \times 10^3$ | 2/10 | 30 | 4/10 | 30 |
| FBJ 2/4–2/5 | FBJ 2/6 | $1 \times 10^4$ | 2/7 | 30 | 7/10 | 30 |
| FBJ 3/2–3/4 | FBJ 3/5 | $1 \times 10^5$ | 9/9 | 18 | 8/8 | 18 |
| FBJ 3/5–3/6 | FBJ 3/7 | $1 \times 10^4$ | 4/10 | 17 | 8/8 | 31 |
| FBJ 3/3–3/4 | FBJ 3/6 | $5 \times 10^3$ | 2/10 | 23 | 7/9 | 23 |
| FBJ 3/5–3/6 | FBJ 3/7 | $1 \times 10^3$ | 0/10 | — | 1/8 | 40 |
| FBJ 4/7–4/9 | FBJ 4/10 | $1 \times 10^4$ | 2/9 | 32 | 7/9 | 32 |
| FBJ 5/3–5/4 | FBJ 5/4 | $1 \times 10^5$ | 5/9 | 19 | 5/5 | 19 |
| FBJ 5/4–5/6 | FBJ 5/7 | $1 \times 10^4$ | 2/7 | 20 | 5/7 | 20 |
| FBJ 6/4–6/7 | FBJ 6/8 | $1 \times 10^4$ | 5/10 | 20 | 8/10 | 20 |
| FBJ 6/4–6/5 | FBJ 6/6 | $1 \times 10^4$ | 4/8 | 20 | 8/8 | 20 |
| FBJ 7/2–7/4 | FBJ 7/5 | $1 \times 10^5$ | 8/8 | 40 | 9/9 | 40 |
| FBJ 7/6–7/7 | FBJ 7/7 | $1 \times 10^5$ | 1/8 | 37 | 5/5 | 37 |
| FBJ 7/2–7/3 | FBJ 7/4 | $1 \times 10^4$ | 1/4 | 32 | 7/7 | 32 |
| FBJ 7/3–7/5 | FBJ 7/6 | $5 \times 10^3$ | 2/10 | 14 | 5/9 | 14 |
| FBJ 7/3–7/5 | FBJ 7/6 | $1 \times 10^3$ | 0/4 | — | 0/10 | — |
| FBJ 9/8–9/9 | FBJ 9/10 | $1 \times 10^4$ | 7/7 | 20 | 5/6 | 20 |
| FBJ 9/8–9/9 | FBJ 9/10 | $1 \times 10^3$ | 2/8 | 40 | 1/7 | 40 |
| FBJ 9/3–9/5 | FBJ 9/6 | $1 \times 10^3$ | 0/9 | — | 0/9 | — |
| FBJ 16/3–16/5 | FBJ 16/6 | $1 \times 10^4$ | 1/5 | 27 | 4/5 | 27 |

* Mice received 4 bilateral implantations of x-irradiated (15,000 rad) tumour at 10-day intervals.
† Mice received 400 rad x-irradiation 24 hours before challenge.
‡ Time in days to first palpable tumour.

TABLE II.—*Induction of Host Resistance to FBJ-MSV Sarcoma Cells by Immunization with Identical and Independently-induced FBJ-MSV Tumours*

| Immunizing tumour and transplant generation* | Challenge tumour and transplant generation | Challenge dose† | Tumour outgrowth in: Treated mice | Latent period† | Untreated controls | Latent period‡ |
|---|---|---|---|---|---|---|
| FBJ 2/11–2/12 | FBJ 4/9 | $1 \times 10^4$ | 4/9 | 30 | 9/10 | 26 |
| FBJ 2/4–2/5 | FBJ 2/6 | $1 \times 10^4$ | 2/7 | 30 | 7/10 | 30 |
| FBJ 3/9–3/11 | FBJ 7/8 | $1 \times 10^4$ | 4/5 | 27 | 5/5 | 27 |
| FBJ 3/5–3/6 | FBJ 3/7 | $1 \times 10^4$ | 4/10 | 17 | 8/8 | 17 |
| FBJ 3/9–3/11 | FBJ 7/8 | $5 \times 10^3$ | 2/5 | 27 | 5/5 | 27 |
| FBJ 3/3–3/4 | FBJ 3/6 | $5 \times 10^3$ | 2/10 | 23 | 7/9 | 23 |
| FBJ 4/2–4/4 | FBJ 2/4 | $1 \times 10^4$ | 5/9 | 25 | 10/10 | 25 |
| FBJ 4/7–4/9 | FBJ 4/10 | $1 \times 10^4$ | 2/9 | 32 | 7/9 | 32 |
| FBJ 4/9–4/7 | FBJ 10/9 | $1 \times 10^4$ | 0/5 | — | 4/9 | 30 |
| FBJ 6/6–6/7 | FBJ 3/8 | $1 \times 10^4$ | 2/5 | 20 | 7/10 | 20 |
| FBJ 6/10–6/12 | FBJ 4/10 | $2 \times 10^4$ | 4/9 | 24 | 5/5 | 24 |
| FBJ 6/6–6/7 | FBJ 4/10 | $1 \times 10^4$ | 0/7 | 24 | 8/10 | 24 |
| FBJ 6/4–6/7 | FBJ 6/8 | $1 \times 10^4$ | 5/10 | 20 | 8/10 | 20 |
| FBJ 7/9–7/10 | FBJ 1/11 | $1 \times 10^4$ | 0/5 | 20 | 4/9 | 20 |
| FBJ 7/9–7/10 | FBJ 3/11 | $1 \times 10^4$ | 2/6 | 16 | 5/7 | 16 |
| FBJ 7/10–7/12 | FBJ 4/9 | $1 \times 10^4$ | 1/6 | 21 | 5/5 | 21 |
| FBJ 7/10–7/12 | FBJ 4/9 | $1 \times 10^4$ | 1/5 | 21 | 6/10 | 21 |
| FBJ 7/2–7/3 | FBJ 7/4 | $1 \times 10^4$ | 1/4 | 32 | 7/7 | 32 |
| FBJ 7/10–7/12 | FBJ 19/6 | $2 \times 10^4$ | 2/5 | 27 | 4/5 | 27 |
| FBJ 7/10–7/12 | FBJ 2/12 | $1 \times 10^4$ | 3/8 | 30 | 7/8 | 30 |
| FBJ 7/10–7/12 | FBJ 6/10 | $1 \times 10^4$ | 4/9 | 23 | 9/9 | 23 |

* Mice received 4 bilateral implantations of x-irradiated (15,000 rad) tumour at 10-day intervals.
† Mice received 400 rad x-irradiation 24 hours before challenge.
‡ Time in days to first palpable tumour.

combinations, immunity was induced by 5 FBJ tumours against challenge with 7 different FBJ sarcomata (Table II). Quantitatively, the resistance induced was comparable with that obtained in mice immunized and challenged with identical tumours. In only one example, where mice were immunized with FBJ 3 and challenged with FBJ 7, were differences in tumour incidences in test and control groups insignificant.

To establish the specificity of these cross-reacting antigens for sarcomata induced by FBJ-MSV, and to eliminate the possibility that resistance was due to a nonspecific increase in immune responsiveness, mice variously received irradiated grafts of normal syngeneic and allogeneic tissues or of syngeneic sarcomata of putatively nonviral origin (Table III), known from comparable transplantation tests to possess TSTA. Thereafter they were challenged with FBJ-MSV tumours at inocula comparable with those at which resistance to the latter was consistently induced. In 11 experiments in which mice were pretreated with normal syngeneic or allogeneic tissues and challenged with 5 different FBJ-MSV sarcomata, neither resistance nor a significant delay in tumour outgrowth could be demonstrated. Similar inability to protect against challenge with FBJ-MSV sarcoma cells was demonstrated for 3 antigenically distinctive sarcomata (MCB2, MCB3 and S115), an observation which was confirmed in a reciprocal test where preimmunization with FBJ 7 failed to protect against challenge with S115, a weakly antigenic radiation-induced osteosarcoma (Moore and Williams, 1972).

(b) *Response to tumour excision*

The immune response evoked by FBJ-MSV induced sarcomata was also determined by excision of subcutaneously developing tumour grafts. By previously established criteria of tumour resistance. in a series of 8 tests 7 tumours exhibited significant immunogenicity. Tumour in-

TABLE III.—*Specificity of Host Resistance Induced by Irradiated Isografts of FJB-MSV Induced Murine Sarcomata*

| Immunizing tumour and transplant generation* | Challenge tumour and transplant generation | Challenge dose† | Tumour outgrowth in: Treated mice | Latent period‡ | Untreated controls | Latent period‡ |
|---|---|---|---|---|---|---|
| FBJ 2/4–2/5 | FBJ 2/7 | $1\times10^4$ | 1/6 | 30 | 7/10 | 30 |
| MCB 3/5–3/9 | FBJ 2/7 | $1\times10^4$ | 11/11 | 32 | 7/7 | 32 |
| CBA(H) | | | | | | |
| Normal tissue | FBJ 2/7 | $1\times10^4$ | 8/10 | 30 | 8/10 | 30 |
| FBJ 7/6–7/8 | FBJ 7/9 | $1\times10^4$ | 1/5 | 30 | 7/7 | 30 |
| MCA 2/4–2/6 | FBJ 7/9 | $1\times10^4$ | 9/9 | 30 | 8/9 | 30 |
| CBA (H) | | | | | | |
| Normal tissue | FBJ 7/10 | $1\times10^4$ | 10/10 | 30 | 8/8 | 30 |
| FBJ 7/5–7/8 | S 115/14 | $1\times10^3$ | 9/9 | 17 | 7/8 | 17 |
| S115/16–S115/18 | FBJ 6/6 | $5\times10^3$ | 9/9 | 21 | 6/6 | 21 |
| CBA (H) | | | | | | |
| Normal tissue | FBJ 1/10 | $1\times10^4$ | 6/9 | 42 | 6/10 | 42 |
| FBJ 1/10–1/12 | FBJ 1/12 | $1\times10^4$ | 1/10 | 42 | 4/10 | 30 |
| CBA(H) | | | | | | |
| Normal tissue | FBJ 7/11 | $1\times10^4$ | 5/5 | 26 | 5/5 | 26 |
| FBJ 7/2–7/3 | FBJ 7/4 | $1\times10^4$ | 1/4 | 32 | 7/7 | 32 |
| CBA(H) | | | | | | |
| Normal tissue | FBJ 7/11 | $5\times10^3$ | 5/6 | 30 | 5/7 | 30 |
| FBJ 7/3–7/5 | FBJ 7/6 | $5\times10^3$ | 2/10 | 14 | 5/9 | 14 |
| CBA(H) | | | | | | |
| Normal tissue | FBJ 3/10 | $1\times10^4$ | 8/10 | 21 | 8/10 | 21 |
| FBJ 3/5–3/6 | FBJ 3/7 | $1\times10^4$ | 4/10 | 17 | 8/8 | 17 |
| BALB/c | | | | | | |
| Normal tissue | FBJ 7/10 | $1\times10^4$ | 9/9 | 25 | 5/6 | 25 |
| CBA(H) | | | | | | |
| Normal tissue | FBJ 7/10 | $1\times10^4$ | 4/5 | 25 | 5/5 | 25 |
| FBJ 7/2–7/3 | FBJ 7/4 | $1\times10^4$ | 1/4 | 32 | 7/2 | 32 |
| BALB/c | | | | | | |
| Normal tissue | FBJ 6/10 | $1\times10^4$ | 6/6 | 21 | 6/6 | 21 |
| CBA(H) | | | | | | |
| Normal tissue | FBJ 6/10 | $1\times10^4$ | 6/6 | 21 | 6/6 | 21 |
| FBJ 7/2–7/3 | FBJ 6/8 | $1\times10^4$ | 5/10 | 20 | 8/10 | 20 |

MCA2—Methylcholanthrene-induced sarcoma in C3H mouse.
MCB3—Methylcholanthrene-induced sarcoma in CBA(H) mouse.
S115 Radiation induced sarcoma in CBA(H) mouse.
* Mice received 4 bilateral implantations of x-irradiated (15,000 rad) tumour at 10-day intervals.
† Mice received 400 rad x-irradiation 24 hours before challenge.
‡ Time in days to first palpable tumour.

cidences following challenge with $10^4$ cells in mice previously exposed to a growing neoplasm were invariably lower than in non-immune controls (Table IV). Tumour outgrowth in mice immunized by this procedure did not differ significantly from that observed following immunization with irradiated isografts nor was there any apparent difference in the level of host resistance (Tables I and II).

Antigenic cross-reactivity of the FBJ-MSV sarcomata was again demonstrated by this technique. Thus, in 4 independent tests in which mice immunized with 5 FBJ-MSV tumours were cross-challenged with 3 different sarcomata, resistance was readily demonstrable.

In a further 4 experiments mice subjected to mock excision or from which developing syngeneic embryonic tissue was excised did not prove resistant to challenge with 2 FBJ-MSV tumours. Excision of an antigenically unrelated neoplasm (MCB 2) likewise failed to protect. Consistent with results obtained by the method of immunization with irradiated tumour grafts, these experiments underlined the specificity of the immune response to FBJ-MSV induced sarcomata.

TABLE IV.—*Host Resistance Following Excision of Subcutaneous Transplants of Murine Sarcomata Induced by FBJ-MSV*

| Immunizing tumour and transplant generation* | Challenge tumour and transplant generation | Challenge dose† | Tumour outgrowth in: Treated mice | Latent period‡ | Untreated controls | Latent period‡ |
|---|---|---|---|---|---|---|
| FBJ 1/13 | FBJ 1/13 | $1\times10^4$ | 1/7 | 29 | 7/8 | 29 |
| FBJ 1/13 | FBJ 4/10 | $1\times10^4$ | 1/6 | 34 | 6/8 | 34 |
| FBJ 2/4 | FBJ 2/6 | $1\times10^4$ | 2/7 | 34 | 7/10 | 34 |
| FBJ 2/4 | FBJ 7/10 | $1\times10^4$ | 2/6 | 40 | 10/10 | 40 |
| FBJ 3/9 | FBJ 3/9 | $1\times10^4$ | 3/5 | 42 | 6/6 | 42 |
| FBJ 4/2 | FBJ 4/3 | $1\times10^4$ | 1/6 | 37 | 8/10 | 37 |
| FBJ 4/6 | FBJ 5/6 | $1\times10^5$ | 6/9 | 22 | 8/8 | 22 |
| FBJ 5/6 | FBJ 7/10 | $1\times10^4$ | 2/7 | 36 | 9/10 | 36 |
| FBJ 6/14 | FBJ 6/14 | $1\times10^4$ | 4/8 | 40 | 10/10 | 40 |
| FBJ 7/10 | FBJ 7/10 | $1\times10^4$ | 2/5 | 31 | 10/10 | 31 |
| FBJ 7/10 | FBJ 7/10 | $1\times10^4$ | 4/9 | 33 | 9/10 | 33 |
| FBJ 7/10 | FBJ 5/6 | $1\times10^5$ | 5/8 | 21 | 8/9 | 21 |
| Mock excision | FBJ 2/6 | $1\times10^4$ | 5/6 | 29 | 6/6 | 29 |
| Mock excision | FBJ 7/10 | $1\times10^4$ | 6/6 | 32 | 6/6 | 32 |
| Syngeneic embryoma | FBJ 7/10 | $1\times10^4$ | 8/8 | 37 | 8/8 | 37 |
| MCB 2/4 | FBJ 7/9 | $1\times10^4$ | 8/8 | 30 | 8/8 | 30 |

MCB2—Methylcholanthrene induced sarcoma in CBA(H) mouse.
* Mice received four bilateral implantations of x-irradiated (15,000 rad) tumour at 10-day intervals.
† Mice received 400 rad pre-irradiation 24 hours before challenge.
‡ Time in days to first palpable tumour.

(c) *Response to cell-free preparations of FBJ-MSV*

The ability of repeated intraperitoneal immunization with cell-free preparations of known oncogenic potential from FBJ virus-induced sarcomata to protect against outgrowth of viable syngeneic tumour cell inocula was assayed in 6 experiments involving challenge with viable cells from 3 FBJ sarcomata and at inocula of $5\times10^3$ and $10^4$ cells (Table V).

Of these 6 groups, 4 showed a marginal reduction in takes by comparison with untreated controls at both challenge inocula and one demonstrated apparent enhancement of tumour outgrowth. In the remaining group no growth inhibition was observed as a result of preimmunization with cell-free FBJ-MSV tumour preparations. The time at which tumours were first palpable (latent period) did not differ in pre-treated and untreated mice.

These experiments underlined the relative inefficiency of cell-free extracts of FBJ-MSV sarcomata to induce immunity compared with procedures involving exposure of the host to intact tumour cells.

TABLE V.—*Host Resistance Following Intraperitoneal Inoculation of Oncogenic Cell-free Preparations of FBJ-MSV*

| No. of injections of FBJ-MSV† | Challenge tumour and transplant generation | Challenge dose* | Tumour outgrowth in: Treated mice | Latent period‡ | Untreated controls | Latent period‡ | Primary tumour incidence in neonates injected with immunizing preparation§ |
|---|---|---|---|---|---|---|---|
| 6 | FBJ 7/11 | $10^4$ | 8/8 | 30 | 8/9 | 30 | 1/5 |
| 6 | FBJ 7/11 | $10^4$ | 3/5 | 27 | 4/5 | 27 | 3/7 |
| 5 | FBJ 7/14 | $5\times10^3$ | 1/4 | 27 | 3/5 | 27 | 2/6 |
| 5 | FBJ 3/10 | $10^4$ | 3/5 | 27 | 4/4 | 27 | 4/8 |
| 4 | FBJ 3/13 | $5\times10^3$ | 5/6 | 32 | 3/6 | 32 | 2/7 |
| 5 | FBJ 2/12 | $5\times10^3$ | 1/5 | 26 | 3/5 | 26 | |

* Mice received 400 rad x-irradiation 24 hours before challenge.
† Mice received i.p. injections (0·5 ml) of cell-free extract at 10-day intervals.
‡ Time in days to first palpable tumour.
§ Fraction of AKR mice yielding sarcomata within 90 days of injection as newborns with 0·1 ml of immunizing virus preparation.

(d) *Neutralization of viable FBJ-MSV tumour cells*

The capacity of cell preparations obtained from the peritoneal cavity and spleen of hyperimmunized mice to effect a reduction in tumour incidence when admixed with viable tumour cells was assayed in 19 experiments (Table VI).

Spleen and peritoneal exudate cells from 8 pre-immunized donors were tested in various combinations against 5 FBJ-MSV sarcoma cell suspensions at a constant effector cell : target cell ratio of 20 : 1. In all but one example (FBJ 13) when target cells alone were injected subcutaneously into pre-irradiated (400 rad) recipient mice, tumour outgrowth occurred in all animals. Suppression of tumour growth was sometimes observed with peritoneal exudate and spleen cell populations from normal mice or mice immunized against an antigenically unrelated tumour (MCB 2). However, the percentage reduction in tumour takes over tumour cells alone in these instances did not exceed 25%. By contrast, spleen and peritoneal exudate cells from mice pre-immunized against FBJ-MSV sarcoma cells consistently reduced the number of tumour takes in pre-irradiated recipients. This effect was observed regardless of whether effector cells were derived from mice immunized with the same FBJ-MSV tumour as the target cell or from mice immune to different FBJ-MSV induced sarcomata.

No significant differences were observed in the time of appearance of first palpable tumours (latent period) in groups which received tumour cells alone, or tumour target cells admixed with effector cells from previously immunized mice or their normal untreated counterparts.

### DISCUSSION

In studies on the immunology of tumour cells transformed by the murine sarcoma viruses (MSV), most attention to date has focussed on sarcomata induced by MSV-M (Moloney) and MSV-H (Harvey), both of which belong to the FMRGr subgroup of oncornavirus type specificity (Fefer *et al.*, 1967*a*; Chuat *et al.*, 1969). These viruses rapidly induce neoplastic and non-neoplastic lesions in a variety of host species (Harvey and East, 1971). The absence of such non-neoplastic conditions in association with the host–virus interaction in mice infected with FBJ-MSV (Finkel *et al.*, 1966; Price *et al.*, 1972) and the wild-type antigenic specificity (Kelloff *et al.*, 1969) distinguish FBJ-MSV induced sarcomata from those induced by MSV isolates of the FMRGr subgroup and justify investigation of the immunological properties of this tumour–host system.

In common with experimental neoplasms induced by oncogenic DNA and RNA viruses, tumour cells transformed *in vivo* by FBJ-MSV possess cell surface antigens capable of evoking tumour rejection responses in syngeneic hosts. Specific resistance to transplanted inocula of FBJ-MSV sarcoma cells could be built up by pretreatment of adult hosts with x-irradiated tumour cells or by excision of subcutaneously developing tumour grafts. The ability of lymphoid cell populations from specifically immune donors to inhibit outgrowth *in vivo* of FBJ-MSV sarcoma cells transplanted to syngeneic recipients provided additional evidence for the antigenicity of these neoplasms. Furthermore, the induction of transplantation resistance is paralleled by the appearance of humoral antibodies in the serum of immunized mice reactive with cell-surface antigens of FBJ-MSV cells and detectable by indirect immunofluorescence tests on viable cell suspensions (to be published).

Transplantation tests involving immunization and challenge with independently induced FBJ-MSV sarcomata established that these tumours possess overlapping antigenic specificities comparable with those shared by other virus-induced neoplasms. Similar results were obtained by neutralization tests utilizing FBJ-MSV target cells and lym-

TABLE VI.—*Inhibition of Outgrowth of FBJ-MSV Sarcoma Cells* in vivo *Following* in vitro *Incubation with Lymphoid Cells from Immune Donors*

| Source of effector cells | Effector cell type | Target* cell | Tumour outgrowth in group | % Inhibition of outgrowth *in vivo* over target cells alone‡ |
|---|---|---|---|---|
| FBJ 1 immune | Spleen | FBJ 4/7 | 1/5 | 80 |
| Normal | Spleen | FBJ 4/7 | 4/5 | 20 |
| FBJ 1 immune | Peritoneal exudate | FBJ 4/7 | 4/5 | 20 |
| Normal | Peritoneal exudate | FBJ 4/7 | 4/4 | 0 |
| — | — | FBJ 4/7 | 4/4 | — |
| FBJ 7/11 immune | Peritoneal exudate | FBJ 7/12 | 1/5 | 80 |
| Normal donor | Peritoneal exudate | FBJ 7/12 | 4/5 | 20 |
| MCB-2† immune | Peritoneal exudate | FBJ 7/12 | 4/5 | 20 |
| — | — | FBJ 7/12 | 5/5 | — |
| FBJ 711 immune | Peritoneal exudate | FBJ 7/12 | 3/5 | 40 |
| FBJ 2/14 immune | Peritoneal exudate | FBJ 7/12 | 3/5 | 40 |
| Normal donor | Peritoneal exudate | FBJ 7/12 | 5/5 | 0 |
| FBJ 7/11 immune | Spleen | FBJ 7/12 | 2/5 | 60 |
| FBJ 2/14 immune | Spleen | FBJ 7/12 | 2/5 | 60 |
| Normal donor | Spleen | FBJ 7/12 | 5/5 | 0 |
| FBJ 13/9 immune | Spleen | FBJ 13/9 | 0/4 | 100 |
| Normal donor | Spleen | FBJ 13/9 | 3/5 | 25 |
| FBJ 13/9 immune | Peritoneal exudate | FBJ 13/9 | 2/5 | 50 |
| Normal donor | Peritoneal exudate | FBJ 13/9 | 4/5 | 0 |
| FBJ 11/7 immune | Spleen | FBJ 13/9 | 0/4 | 100 |
| Normal donor | Spleen | FBJ 13/9 | 3/5 | 25 |
| FBJ 11/7 immune | Peritoneal exudate | FBJ 13/9 | 1/5 | 75 |
| Normal donor | Peritoneal exudate | FBJ 13/9 | 4/5 | 0 |
| — | — | FBJ 13/9 | 4/5 | — |
| FBJ 19/6 immune | Spleen | FBJ 19/6 | 3/5 | 40 |
| Normal donor | Spleen | FBJ 19/6 | 5/5 | 0 |
| FBJ 13/10 immune | Spleen | FBJ 19/6 | 2/5 | 60 |
| MCB-2 immune | Spleen | FBJ 19/6 | 4/5 | 20 |
| — | — | FBJ 19/6 | 5/5 | — |
| FBJ 21/6 immune | Peritoneal exudate | FBJ 21/7 | 3/5 | 40 |
| Normal donor | Peritoneal exudate | FBJ 21/7 | 4/5 | 20 |
| FBJ 21/6 immune | Spleen | FBJ 21/7 | 1/5 | 80 |
| Normal donor | Spleen | FBJ 21/7 | 4/5 | 20 |
| — | — | FBJ 21/7 | 5/5 | — |

* Effector cell : target cell ratio 20 : 1 in all cases.
† MCB-2 methylcholanthrene induced sarcoma.
‡ Standard target cell inoculum $5 \times 10^4$ cells.

phoid cells from hosts immunized against different FBJ-MSV induced sarcomata. These specificities were not present on normal adult host tissues of the CBA(H) strain, on certain allogeneic tissues, nor on murine sarcomata induced by other aetiological agents, *e.g.* chemical carcinogens and radiation. In respect of antigenicity in syngeneic hosts, tumours induced by FBJ-MSV are thus similar to those transformed by MSV isolates including MSV-H (Chuat *et al.*, 1969; Koldovsky *et al.*, 1969) MSV-M (Fefer *et al.*, 1967*a*, *b*, *c*; 1968; Law *et al.*, 1968; Stanton, Law and Ting, 1968) and MSV-K (Kirsten) (McCoy *et al.*, 1972).

The nature of the FBJ-MSV tumour-rejection antigen(s) cannot be elucidated at present. Transplantation methodology as employed in this investigation is incapable of establishing whether the rejection phenomenon in this tumour–host system is mediated primarily by new virus-determined cellular antigens or, since FBJ-MSV cells release infectious virus, by components of the viral capsid incorporated into the cell surface membrane. Among neoplasms of the murine leukaemia–sarcoma virus complex, the presence of mature virions in tumours has complicated attempts to discriminate between cellular and virion antigens expressed by transformed cells. The coexistence of lesions such as atypical granulomata, erythroblastic splenomegaly and disseminated cystic lesions among MSV isolates of the FMRGr subgroup are indicative of host–virus interactions additional to the process of neoplastic transformation (Harvey and East, 1971). Within these systems, immunization with cell-free virus preparations is effective in protecting against challenge with tumour cell inocula (Chuat *et al.*, 1969; Koldovsky *et al.*, 1969; Fink and Rauscher, 1964; Kobayashi and Takeda, 1967) and it appears that the rejection response is mediated principally by viral antigens present on the cell surface (Law, 1970). This conclusion is supported by the induction of transplantation resistance to MSV-M sarcomata by MSV-M induced lesions which are not strictly neoplastic tissues and by the passive transfer of immunity by anti-MSV antibody (Law *et al.*, 1968).

In this study immunization with intact FBJ-MSV transformed cells was a far more effective and reliable procedure for inducing immunity than repeated inoculation of adults with virus-containing cell free extracts. This might imply that integrity of cell surface structure is obligatory for consistent immunogenicity and suggest that the rejection phenomenon is mediated by a new cell surface antigen either not identifiable as FBJ-MSV itself or present in a modified form, although other explanations are clearly possible. The demonstration of tumour rejection antigens distinct from virion antigens on the surface of virus-transformed cells was claimed by Ting (1967) for a neoplasm (MSB-1) of epithelial cell origin in the rat induced by MSV; and by Law and Ting (1970) for a transplantable murine haemangiosarcoma (XM-1) induced by MSV. However, the former cell line was subsequently found to release both a focus forming virus (MSV-0) (Ting, 1968) and a rat-tropic helper leukaemia virus (Aaronson, 1971) while the latter also produces C-type virus particles. By contrast, Stephenson and Aaronson (1972) showed that MSV-transformed Balb/3T3 non-producer cells lack detectable transplantation antigens and suggest that transplantation resistance to the producing cells is attributable to maturing virus at the cell surface.

The problem of the nature of the FBJ-MSV tumour rejection antigen is, however, further complicated by the fact that, in common with other RNA members of the murine oncogenic leukaemia–sarcoma virus complex, FBJ-MSV is found associated with a non-pathogenic virus (FBJ-MLV) at high titre (Levy, Hartley and Huebner, 1973). At present it is not known if this virus is a non-pathogenic murine leukaemia virus, a non-pathogenic mutant of FBJ-MSV, or whether FBJ-MLV rescued a sarcoma genome from an original CF-1 sarcoma cell and returned FBJ-MSV to an infectious state. Analogous studies on neoplasms of the leukaemia–sarcoma complex in the FMRGr subgroup, where the associated leukaemia viruses are pathogenic and function as helpers for the production of infectious MSV, have failed to reveal differences in antigenic specificity in cells transformed by MSV and MLV (Fefer *et al.*, 1967*a*; Chuat *et al.*, 1969; Strouk *et al.*, 1972). A similar situation might be found to pertain for cells transformed by viruses of the FBJ MSV/MLV complex.

The maximum level of resistance induced by FBJ-MSV cells in syngeneic hosts did not exceed $10^5$ cells, *i.e.* the antigenicity of these neoplasms is of a relatively weak order compared with murine sarcomata induced by classic chemical carcinogens (Klein *et al.*, 1960). It is noteworthy that in this respect FBJ-MSV induced neoplasms are comparable with spontaneous murine neoplasms (Prehn and Main, 1957; Hammond, Fisher and Rolley, 1967) and with virus induced murine leukaemias of the Gross (G) or "wild" type subgroup (Klein *et al.*, 1962; Stettenmark and Klein, 1962) as opposed to leukaemias and sarcomata of the FMRGr subgroups which are much more strongly antigenic (Klein and Klein, 1964; Fefer *et al.*, 1967*a*). This antigenic weakness on the part of the naturally occurring leukaemia–sarcoma viruses, together with the findings on the natural occurrence of Gross virus and the specific cellular antigen (G) determined by it (Old, Boyse and Stockert, 1965; Aoki *et al.*, 1966), suggest that the agents may reflect a multivalent virus having a different potential in different host cells or a heterogeneous population of pathogenic and non-pathogenic variants present in nature. By contrast, this situation is not necessarily true of some of the other viral agents of the FMRGr subgroup which were isolated from serially transplanted neoplasms in the laboratory and which induce highly antigenic neoplasms less likely to occur under natural circumstances.

We thank Dr N. W. Nisbet, Director of Research, for his support and Miss Janet Hotchkiss for secretarial assistance. This study was supported by grants from the Cancer Research Campaign and the Medical Research Council.

### REFERENCES

Aaronson, S. A. (1971) Isolation of a Rat-tropic Helper Virus from M-MSV-O Stocks. *Virology*, **44**, 29.

Aoki, T., Boyse, E. A. & Old, L. J. (1966) Occurrence of Natural Antibody to the Gross (G) Leukaemia Antigen in Mice. *Cancer Res.*, **26**, 1415.

Chuat, J-C., Berman, L., Gunvén, P. & Klein, E. (1969) Studies on Murine Sarcoma Virus: Antigenic Characterization of Murine Sarcoma Virus Induced Tumour Cells. *Int. J. Cancer*, **4**, 465.

Fefer, A., McCoy, J. L. & Glynn, J. P. (1967*a*) Antigenicity of a Virus-induced Murine Sarcoma (Moloney). *Cancer Res.*, **27**, 962.

Fefer, A., McCoy, J. L. & Glynn, J. P. (1967*b*) Induction and Regression of Primary Moloney Sarcoma Virus-induced Tumors in Mice. *Cancer Res.*, **27**, 1626.

Fefer, A., McCoy, J. L. & Glynn, J. P. (1967*c*) Studies on the Growth and Regression of Transplantable Moloney Sarcoma. *Cancer Res.*, **27**, 2207.

Fefer, A., McCoy, J. L., Perk, K. & Glynn, J. P. (1968) Immunologic, Virologic and Pathologic Studies of Regression of Autochthonous Moloney Sarcoma Virus-induced Tumours in Mice. *Cancer Res.*, **28**, 1577.

Fink, M. A. & Rauscher, F. J. (1964) Immune Reactions to a Murine Leukaemia Virus. 1. Induction of Immunity to Infection with Virus in the Natural Host. *J. natn. Cancer Inst.*, **32**, 1075.

Finkel, M. P., Biskis, B. O. & Jinkins, P. B. (1966) Virus Induction of Osteosarcomas in Mice. *Science, N.Y.*, **151**, 698.

Globerson, A. & Feldman, M. (1964) Antigenic Specificity of Benzo-pyrene-induced Sarcomas. *J. natn. Cancer Inst.*, **32**, 1229.

Glynn, J. P., Bianco, A. R. & Goldin, A. (1964) Studies on Induced Resistance against Isotransplants of Virus-induced Leukaemia. *Cancer Res.*, **24**, 502.

Hammond, W. G., Fisher, J. C. & Rolley, R. T. (1967) Tumour-specific Transplantation Immunity to Spontaneous Mouse Tumours. *Surgery, St Louis*, **62**, 124.

Harvey, J. J. (1964) An Unidentified Virus which Causes the Rapid Production of Tumours in Mice. *Nature, Lond.*, **204**, 1104.

Harvey, J. J. & East, J. (1971) The Murine Sarcoma Virus (MSV) *Int. Rev. exp. Path.*, **10**, 265.

Kelloff, G. J., Lane, W. T., Turner, H. C. & Huebner, R. J. (1969) *In vivo* Studies of the FBJ Murine Osteosarcoma Virus. *Nature, Lond.*, **223**, 1380.

Klein, G. (1968) Tumour-specific Transplantation Antigens: G. H. A. Clowes Memorial Lecture. *Cancer Res.*, **28**, 625.

Klein, E. & Klein, G. (1964) Antigenic Properties of Lymphomas Induced by the Moloney Agent. *J. natn. Cancer Inst.*, **32**, 547.

Klein, G., Sjögren, H. O. & Klein, E. (1962) Demonstration of Host Resistance against Isotransplantation of Lymphomas Induced by the Gross Agent. *Cancer Res.*, **22**, 955.

Klein, G., Sjögren, H. O., Klein, E. & Hellström, K. E. (1960) Demonstration of Resistance against Methyl-cholanthrene-induced Sarcomas in the Primary Autochthonous Host. *Cancer Res.*, **20**, 1561.

Kobayashi, H. & Takeda, H. (1967) Inhibition of the Growth of Friend Tumour Cell in Mice Immunised with Live Friend Virus. *Gann*, **58**, 25.

Koldovsky, P., Turano, A. & Fadda, G. (1969) Specific Transplantation Resistance against Mouse Tumour Induced by Mouse Sarcoma Virus Harvey. *Folia Biol., Praha*, **15**, 224.

Law, L. W. (1969) Studies on the Significance of Tumour Antigens in Induction and Regression of Neoplastic Diseases. *Cancer Res.*, **29**, 1.

Law, L. W. (1970) Studies of Tumour Antigens and Tumour-specific Immune Mechanisms in Experimental Systems. *Transplantn Proc.*, **2**, 117.

Law, L. W. & Ting, R. C. (1970) Antigenic Properties of a Non-releaser Neoplasm Induced in the Mouse by Murine Sarcoma Virus. *J. natn. Cancer Inst.*, **44**, 615.

Law, L. W., Ting, R. C. & Stanton, M. F. (1968) Some Biologic, Immunogenic and Morphologic Effects in Mice after Infection with a Murine Sarcoma Virus. I. Biologic and Immunogenic Studies. *J. natn. Cancer Inst.*, **40**, 1101.

Levy, J. A., Hartley, J. W. & Huebner, R. J. (1973) Studies of FBJ Osteosarcoma Virus in Tissue culture. I. Biologic Characteristics of the " C " Type Viruses. In the press.

McCoy, J. L., Fefer, A., McCoy, N. T. & Kirsten, W. H. (1972) Immunobiological Studies of Tumours Induced by Murine Sarcoma Virus (Kirsten). *Cancer Res.*, **32**, 343.

Moore, M. & Williams, D. E. (1972) Studies on the Antigenicity of Radiation-induced Murine Osteosarcomata. *Br. J. Cancer*, **26**, 90.

Old, L. J. & Boyse, E. A. (1964) Immunology of Experimental Tumours. *A. Rev. Med.*, **15**, 167.

Old, L. J., Boyse, E. A. & Lilly, F. (1963) Formation of Cytotoxic Antibody against Leukaemias Induced by Friend Virus. *Cancer Res.*, **23**, 1063.

Old, L. J., Boyse, E. A. & Stockert, E. (1964) Typing of Mouse Leukaemias by Serological Methods. *Nature, Lond.*, **201**, 777.

Old, L. J., Boyse, E. A. & Stockert, E. (1965) The G (Gross) Leukaemia Antigen. *Cancer Res.*, **25**, 813.

Pasternak, G. & Hölzer, B. (1965) Der nachweis von Immunologischen differenzen zwischen Graffi- und Gross-virus-induzierten Leukamien der maus *in vivo* und *in vitro*. *Neoplasma*, **12**, 339.

Perk, K. & Moloney, J. B. (1966) Pathogenesis of a Virus Induced Rhabdomyosarcoma in Mice. *J. natn. Cancer Inst.*, **37**, 581.

Prehn, R. T. & Main, J. M. (1957) Immunity to Methylcholanthrene-induced Sarcomas. *J. natn. Cancer Inst.*, **18**, 769.

Price, C. H. G., Moore, M. & Jones, D. B. (1972) FBJ Virus-induced Tumours in Mice. A Histopathological Study of FBJ Virus Tumours and their Relevance to Murine and Human Osteosarcoma Arising in Bone. *Br. J. Cancer*, **26**, 15.

Sjögren, H. O. (1964) Studies on Specific Transplantation Resistance to Polyoma Virus-induced Tumours. IV. Stability of the Polyoma Antigen. *J. natn. Cancer Inst.*, **32**, 661.

Sjögren, H. O. (1965) Transplantation Methods as a Tool for Detection of Tumor-specific Antigens. *Prog. exp. Tumor Res.*, **6**, 289.

Slettenmark, B. & Klein, E. (1962) Cytotoxic and Neutralisation Tests with Serum and Lymph Node Cells of Isologous Mice with Induced Resistance against Gross Lymphomas. *Cancer Res.*, **22**, 947.

Stanton, M. F., Law, L. W. & Ting, R. C. (1968) Some Biologic, Immunogenic and Morphologic Effects in Mice after Infection with a Murine Sarcoma Virus. II. Morphologic Studies. *J. natn. Cancer Inst.*, **40**, 113.

Stephenson, J. R. & Aaronson, S. A. (1972) Antigenic Properties of Murine Sarcoma Virus-transformed BALB/3T3 Non-producer Cells. *J. exp. Med.*, **135**, 503.

Strouk, V., Grundner, G., Fenyo, E. M., Lamon, E., Skurzak, H. & Klein, G. (1972) Lack of Distinctive Surface Antigen on Cells Transformed by Murine Sarcoma Virus. *J. exp. Med.*, **136**, 344.

Ting, R. C. (1967) Tumor Induction in Thymectomized Rats by Murine Sarcoma Virus (Moloney) and Properties of the Induced Virus-free Tumor Cells. *Proc. exp. Biol. Med.*, **126**, 778.

Ting, R. C. (1968) Biological and Serological Properties of Viral Particles from a Non-producer Rat Neoplasm Induced by Murine Sarcoma Virus (Moloney). *J. Virol.*, **2**, 865.

Wahren, B. (1963) Cytotoxic Assays and Other Immunologic Studies of Leukaemias Induced by Friend Virus. *J. natn. Cancer Inst.*, **31**, 411.

Yumoto, T., Poel, W. E., Kodama, T. & Dmochowski, L. (1970) Studies on the FBJ Virus-induced Bone Tumours in Mice. *Tex. Rep. Biol. Med.*, **28**, 145.